# The association between parental physical activity and the parent–child relationship in early childhood: the serial mediating roles of emotion regulation and subjective wellbeing

**DOI:** 10.3389/fpsyg.2026.1771516

**Published:** 2026-10-16

**Authors:** Xiaogang Zhang, Keyong Wang, Junmin Zhang, Shuiyou Hu, Jianqiang Tian

**Affiliations:** 1 School of Football Sports, Chengdu Sport University, Chengdu, Sichuan, China; 2 School of Sport and Health Sciences, Sichuan Technology and Business University, Meishan, Sichuan, China

**Keywords:** emotion regulation, parent–child relationship, parents of young children, physical activity, serial mediation, subjective wellbeing

## Abstract

**Objective:**

The parent–child relationship in early childhood is closely linked to children's physical and psychological development. Although parental physical activity has been associated with favorable psychological outcomes, little is known about how it relates to the parent–child relationship or about the potential roles of emotion regulation and subjective wellbeing. Accordingly, this study used structural equation modeling to examine the hypothesized direct and indirect associations among these variables.

**Methods:**

A cross-sectional questionnaire survey was conducted among 719 parents recruited through convenience and snowball sampling in six Chinese cities, including Chengdu, Suining, Meishan, Lanzhou, Xianyang, and Xi'an. All study variables were assessed by the same parent using the Physical Activity Rating Scale, the Cognitive Emotion Regulation Questionnaire, the Brief Subjective Wellbeing Scale for Chinese Citizens, and the Child-Parent Relationship Scale. Data were analyzed using covariance-based structural equation modeling in AMOS 23.0. Complementary PROCESS Model 6 analyses based on observed composite scores were conducted in SPSS 29.0.

**Results:**

(1) Parental physical activity was positively associated with the parent–child relationship, and the direct path between the two variables was statistically significant (β = 0.143, *p* < 0.001). (2) The completely standardized indirect estimate through emotion regulation was 0.041 (95% CI [0.020, 0.065]), while the estimate through subjective wellbeing was 0.050 (95% CI [0.030, 0.074]). The serial indirect estimate was 0.015 (95% CI [0.008, 0.023]).

**Conclusion:**

Higher levels of parental physical activity were associated with higher emotion regulation scores, greater subjective wellbeing, and a more positive parent–child relationship.

## 1 Introduction

The parent–child relationship (PCR) is an interpersonal relationship formed through interactions between caregivers and their children, serving as one of the earliest and most important external resources for children's development (Li et al., 2024). From a physiological perspective, the communication and interaction between young children and their parents are associated with the development and connectivity of neural pathways in the brain, which may contribute to the development of memory, learning, and logical thinking abilities (Shore, 1997). In contrast, insufficient parental care, affection, and communication may be associated with less optimal brain development and increased risks of mental health problems, learning difficulties, and memory impairments (Teicher, 2002). From a psychological perspective, caregivers who are sensitive and responsive to children's needs can not only provide them with a “safe harbor” for exploring the external environment but also offer appropriate guidance and support in times of distress or fear (Ainsworth et al., 2015). Such responsive caregiving also contributes to the development of language skills, cognitive abilities, and executive functions (Rodrigues et al., 2021). Indeed, a positive parent–child relationship has been identified as an important protective factor against children's emotional and behavioral problems, thereby supporting their healthy development (McWey et al., 2015).

Balancing caregiving responsibilities with work and other family obligations can consume substantial amounts of parents' time and psychological resources (Menon et al., 2020), while concerns about children's learning and behavioral development may further intensify their emotional burden (Reijneveld et al., 2008), thereby contributing to parenting stress. Previous studies have shown that parenting stress is associated with less supportive parenting practices and poorer parent–child relationship (Mak et al., 2020; Cai et al., 2021). Against this background, parents' physical activity may buffer the negative effects of parenting stress and support more positive family interactions (Limbers et al., 2020). When parents and children engage in physical activity together, they share an interactive context that is associated with enjoyable experiences, closer family relationships, and children's social development (Filanowski et al., 2021; Koga et al., 2023; Yan et al., 2024). Although this evidence highlights the value of shared physical activity for family interaction, it does not clarify whether parents' own physical activity is related to parent–child relationship. As a healthy lifestyle behavior, physical activity may help parents maintain a positive physical and psychological state, which may be reflected in more positive daily interactions with their children. Therefore, this study aims to examine this relationship and test the hypothesized mediating pathways.

### 1.1 The association between physical activity and the PCR

Physical activity refers to any physical movement that causes energy expenditure through skeletal muscle contraction (Caspersen et al., 1985). It is widely recognized as an effective means of supporting physical and mental health (Fletcher et al., 2018; Kandola et al., 2019). Physical activity may enhance positive feelings through increased endogenous cannabinoids activity (Siebers et al., 2021), improve physical fitness (Khan et al., 2012; Pinho et al., 2024), and be associated with higher levels of momentary happiness (Csikszentmihalyi and Wong, 2014). Although these benefits primarily concern individual health, they may also be relevant to parents' everyday family interactions.

According to family systems theory, the behaviors and emotions of family members can influence one another (Bavelas and Segal, 1982; Iwanski et al., 2022). From this theoretical perspective, the broader benefits of parents' physical activity may also be reflected in daily interactions with their children. Existing evidence indicates that parents' physical activity may reduce the negative impact of parenting stress on social interactions and the family environment (Limbers et al., 2020), foster a more positive parent–child relationship, and support the development of children's social interaction skills (Yan et al., 2024). These findings provide a basis for the following hypothesis.

H1: Parents' own physical activity is positively associated with the quality of the parent–child relationship.

### 1.2 The mediating role of emotion regulation between PA and PCR

Emotion refers to the collection of various psychological states of human beings, including subjective experience, expressive behavior (such as face, limbs, and language), and physiological reactions (such as heart rate and breathing) (Gross and Feldman Barrett, 2011). Emotion regulation (ER) refers to the cognitive processes by which individuals modulate their emotional experiences, encompassing the identification, experience, and expression of emotions (Gross, 1998). Its prominent feature involves actively activating and regulating goals to guide their emotional trajectory (Gross et al., 2011). Emotion regulation can be categorized into two primary types: intrinsic and extrinsic. Intrinsic emotion regulation refers to the regulation process of individuals aiming to adjust their own emotional state, and extrinsic emotion regulation refers to the regulation process of individuals aiming to change the emotional state of others. In reality, a single behavior can have both internal and external regulating functions. For example, parents will avoid losing control of their emotions by soothing crying children (Gross, 2015). Because the present study focuses on how parents regulate their own emotions during parenting, emotion regulation is examined primarily as an intrinsic process and is operationalized through parents' habitual use of cognitive emotion regulation strategies.

Previous studies have demonstrated that parents who are proficient at regulating their emotions are more inclined to adopt positive parenting behaviors, and their children show fewer internalized symptoms (Rutherford et al., 2015; Zimmer-Gembeck et al., 2022). These findings indicate that parents' emotion regulation may be related to the better parent–child relationship. It is worth noting that some studies have shown that a single exercise of more than 30 min can improve emotion regulation, and the effect is not affected by exercise intensity, frequency, and subjects' health status (Liu et al., 2022). Based on this, hypothesis 2 (H2) is proposed in this study.

H2: Emotion regulation plays a mediating role between parents' physical activity and parent–child relationship.

### 1.3 The mediating role of subjective wellbeing between PA and PCR

Subjective wellbeing (SWB) refers to individuals' cognitive and affective evaluations of their lives, typically characterized by high life satisfaction, relatively frequent positive emotions, and relatively infrequent negative emotions (Diener, 1984; Regan et al., 2022). Previous research has indicated that parents' subjective wellbeing is positively related to their children's subjective wellbeing (Casas et al., 2008), suggesting a possible transmission of subjective wellbeing from parents to their children. Beyond its association with children's wellbeing, higher parental subjective wellbeing is associated with more frequent use of effective parenting practices, including warmth, encouragement, limit setting, and shared parent–child activities (da Silva et al., 2023). In contrast, higher parenting stress is associated with lower parental wellbeing (Rusu et al., 2025). Such stress is also linked to reduced patience, poorer parent–child communication, and more punitive or authoritarian parenting (Mirosavljević and Sablić, 2025). Similarly, parental psychological distress is associated with greater use of violent punishment toward young children (Cuartas et al., 2025). These findings suggest that parental subjective wellbeing may be associated with parent–child relationship quality. Furthermore, a meta-analysis confirms a positive relationship between physical activity and subjective wellbeing across different physical activity settings, activity forms, and measurement methods (Buecker et al., 2021). Based on this evidence, hypothesis 3 (H3) is proposed in this study.

H3: Subjective wellbeing plays a mediating role between parents' physical activity and parent–child relationship.

### 1.4 The serial mediating roles of ER and SWB between PA and PCR

As discussed above, parents' physical activity is related to the parent–child relationship, emotion regulation, and subjective wellbeing (Buecker et al., 2021; Liu et al., 2022; Yan et al., 2024). Moreover, parental emotion regulation has been linked to greater satisfaction with the parent–child relationship, while parental subjective wellbeing has been related to greater involvement in shared parent–child activities (Fabrizio et al., 2015; Richter et al., 2018), suggesting that each variable may separately mediate the relationship between parents' physical activity and the parent–child relationship. However, these two proposed mediating pathways may also be sequentially connected. Evidence indicates that the ability to regulate negative emotions is positively related to subjective wellbeing (Tsujimoto et al., 2024). Similar findings have been reported among adolescents and pre-service teachers. Among adolescents, adaptive mood regulation strategies have been linked to higher levels of life satisfaction, happiness, and self-esteem, as well as lower levels of anxiety, depression, and emotional distress (Serey et al., 2025). Among pre-service teachers, emotion regulation was indirectly related to higher subjective wellbeing through psychological resilience (Özdemir et al., 2024). This suggests a possible serial pathway from physical activity to the parent–child relationship through emotion regulation and subjective wellbeing (PA → ER → SWB → PCR). Accordingly, hypothesis 4 (H4) is proposed.

H4: Emotion regulation and subjective wellbeing serially mediate the relationship between parents' physical activity and the parent–child relationship.

In summary, the proposed model specifies parents' physical activity as the independent variable, the parent–child relationship as the dependent variable, and emotion regulation and subjective wellbeing as serial mediators (see Figure 1).

**Figure 1:**
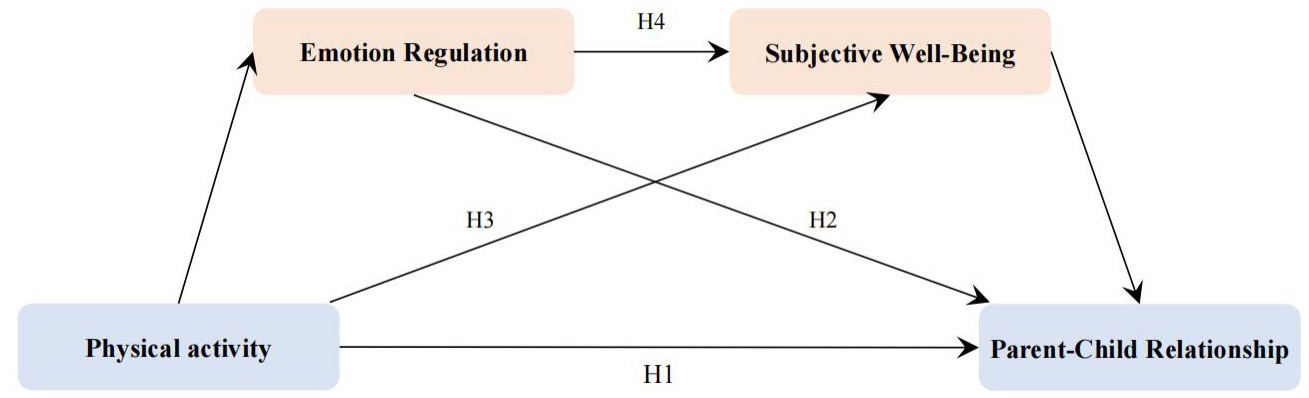
The hypothesized model.

## 2 Method

### 2.1 Participants and procedure

This cross-sectional study used a combination of convenience sampling and snowball sampling to recruit parents of young children in six Chinese cities, including Chengdu, Suining, Meishan, Lanzhou, Xianyang, and Xi'an, as research subjects from September 15 to October 2, 2025. Data were collected through an online questionnaire. During the survey, the researchers explained the purpose and process of the study to the participants, and then the participants filled in the questionnaire independently. At the same time, the study strictly adhered to the principle of voluntary participation and the right to withdraw at any time, ensuring that subjects participate on the basis of informed consent. In the end, 776 responses were received, and 719 valid responses were retained. The inclusion and exclusion criteria are delineated in Table 1.

**Table 1:** Inclusion and exclusion criteria.

Inclusion criteria	Exclusion criteria
(1) being a parent of a child aged 3–6 years	(1) questionnaire completion time less than 120 seconds
(2) voluntary participation in the study	(2) completely identical responses to all items
	(3) patterned responses

Descriptive analysis of the sample showed that 24.2% of participants were male and 75.8% were female. Among parent respondents, 12.2% were under 30 years old (0.7% aged 20–25; 11.5% aged 25–30). The majority were aged 30–35 (43.1%) or over 35 (44.6%). Regarding only-child status, 41.2% had one child, while 58.8% had multiple children. In terms of daily work hours, 14.9% worked ≤ 4 h, 17.4% worked 5–7 h, 49.2% worked 8–9 h, and 18.5% worked >9 h. Finally, the percentages of parents with children in the kindergarten junior class, middle class, and senior class were 27.4%, 31.3%, and 41.3% (see Table 2). Because the 20–25 year age category contained only five participants, it was combined with the adjacent 25–30 year category for the structural analysis.

**Table 2:** Demographic information.

Variable	Category	Frequency	Percentage
Gender	Male	174	24.2%
	Female	545	75.8%
Age	20–25 years	5	0.7%
	25–30 years	83	11.5%
	30–35 years	310	43.1%
	>35 years	321	44.6%
Working hour	≤ 4 h a day	107	14.9%
	5–7 h a day	125	17.4%
	8–9 h a day	354	49.2%
	>9 h a day	133	18.5%
Only child	Yes	296	41.2%
	No	423	58.8%
Child's class	Junior class	197	27.4%
	Middle class	225	31.3%
	Senior class	297	41.3%

The Ethics Review Committee of Chengdu Sport University reviewed and approved this study (approval number: No. [2025] 230).

### 2.2 Measurement tools

#### 2.2.1 Physical activity rating scale

The Chinese version of the Physical Activity Rating Scale (PARS-3) developed by Liang was used to evaluate the physical activity level of parents (Liang, 1994). The three dimensions of the scale are intensity (1–5 points), frequency (1–5 points), and duration (0–4 points). The total physical activity score was calculated as “intensity × frequency × duration” (range: 0–100).

#### 2.2.2 Cognitive emotion regulation questionnaire

A short version of the Cognitive Emotion Regulation Questionnaire developed by Garnefski and Kraaij was used to assess parents' habitual use of cognitive emotion regulation strategies (Garnefski and Kraaij, 2006). This questionnaire comprises 18 items rated on a five-point Likert scale. All items are organized into nine dimensions, each containing two items. The score for each dimension was calculated as the mean of its two corresponding items, and the scores for self-blame, rumination, catastrophizing, and other-blame were reverse-coded so that higher scores across all nine dimensions consistently indicated more adaptive cognitive emotion regulation. Because the hypotheses concerned cognitive emotion regulation at the construct level rather than strategy-specific associations, the nine dimension scores were used as observed indicators of an overall latent cognitive emotion regulation construct. The Cronbach's α of the scale was determined to be 0.954 in the current study.

#### 2.2.3 Brief subjective wellbeing scale for Chinese citizens

The Brief Subjective Wellbeing Scale for Chinese Citizens was compiled by Xing, with a total of 20 questions. Among them, the 4th, 5th, 6th, 9th, 10th, 11th, 13th, 15th, 17th, 18th, and 20th are reverse-scored questions, which need to be converted forward (Xing, 2003). In this study, the scale uses the Likert 5-point scoring method. The 20 item scores were used as observed indicators of the latent construct representing subjective wellbeing. The Cronbach's α of the scale was determined to be 0.966 in the current study.

#### 2.2.4 Child-parent relationship scale

The parent–child relationship was assessed using the Chinese version of the Child-Parent Relationship Scale (CPRS), originally developed by Pianta and translated by Shen (Pianta, 1992; Shen, 2014). This 30-item scale uses the Likert 5-point scoring method (1 = entirely disagree, 5 = entirely agree). Based on the scale's dimensional structure, the three dimensions of Closeness, Conflict, and Dependence served as the observed indicators of the parent–child relationship. As specified in the scale's scoring guide, items 4, 7, 15, and 20 are not assigned to any of these three dimensions and were therefore excluded from the calculation of the dimension scores. Conflict and Dependence scores were reverse-coded so that higher scores on all three indicators represented more positive parent–child relationship quality. The score for each dimension was calculated as the mean of its assigned items. The Cronbach's α of the scale was determined to be 0.977 in the current study.

### 2.3 Statistical analysis

SPSS 29.0 was used to conduct descriptive analyses, assess internal consistency reliability, and examine Pearson correlations among the study variables. AMOS 23.0 was used to implement covariance-based structural equation modeling (CB-SEM), conduct confirmatory factor analysis, evaluate the measurement properties of the latent constructs, and estimate the hypothesized structural equation model. Following the PARS-3 scoring guidelines, exercise intensity, frequency, and duration were multiplied to calculate a total physical activity score, which was entered into the structural equation model as an observed exogenous variable. Emotion regulation, subjective wellbeing, and the parent–child relationship were modeled as latent variables based on their respective observed indicators. Parent gender, age group, daily working hours, only-child status, and the child's kindergarten grade were included as observed covariates. Because only five participants were aged 20–25 years, this category was combined with the 25–30 year category for the adjusted analyses. Age group, daily working hours, and kindergarten grade were represented by dummy variables. The 30–35 year age group, the 8–9 h work category, and junior kindergarten served as the reference groups. Gender was coded 0 for female and 1 for male, while only-child status was coded 0 for no and 1 for yes. Separate paths were estimated from each covariate to emotion regulation, subjective wellbeing, and the parent–child relationship. All exogenous variables were allowed to covary. The total indirect association in the structural model was evaluated using 5,000 bias-corrected bootstrap samples and a 95% confidence interval.

Although AMOS can estimate the total indirect association, its standard output does not automatically provide separate bootstrap confidence intervals for the specific pathways in a serial multiple-mediator model. PROCESS Model 6 was therefore used as a complementary analysis to estimate the ER specific pathway, the SWB specific pathway, and the serial pathway separately based on observed composite scores (Hayes, 2013). The same demographic covariates were entered into the PROCESS model. The mediation analysis followed the established testing procedure (Wen and Ye, 2014). Specific indirect associations were evaluated using 5,000 bootstrap samples and 95% confidence intervals. An indirect association was considered statistically significant when its confidence interval did not include zero. Statistical significance was set at *p* < 0.05. Standardized regression coefficients were used to indicate the magnitude of the associations between variables.

## 3 Results

### 3.1 Reliability and validity assessment

The reliability reflects the stability of the measurement results. Internal consistency reliability is used to detect whether each entry measures the same content or characteristics. It is typically represented by Cronbach's α coefficient. A value above 0.8 indicates a reliable measurement tool (Wu, 2010). Internal consistency reliability was not assessed for the PARS-3 physical activity score because it is calculated by multiplying exercise intensity, frequency, and duration rather than by aggregating a set of homogeneous items. The Cronbach's α coefficients for the instruments measuring emotion regulation, subjective wellbeing, and parent–child relationship were 0.954, 0.966, and 0.977, all of which are higher than the acceptable reliability threshold of 0.8, indicating that the measures have high internal consistency (see Table 3).

**Table 3:** Reliability analysis results.

Dimension	Cronbach's α	Number of items
ER	0.954	18
SWB	0.966	20
PCR	0.977	26

PA, physical activity; ER, emotion regulation; SWB, subjective wellbeing; PCR, parent–child relationship.

All measurement items in this study were drawn from well-established, extensively validated scales. To ensure the accuracy of items that had been translated, one scholar with a doctoral degree in English and four experts in the field of child development critically reviewed all items before the formal survey. All five experts approved the content of the items, indicating good content validity for the scales used in this study.

To evaluate convergent validity, we calculated the composite reliability (CR) and average variance extracted (AVE) from standardized factor loadings. Results indicated that all standardized factor loadings exceeded 0.70, ranging from 0.819 to 0.851 for ER, from 0.705 to 0.834 for SWB, and from 0.940 to 0.971 for PCR. Furthermore, all CR values exceeded 0.7 (ER = 0.954, SWB = 0.966, PCR = 0.973), and all AVE values exceeded 0.5 (ER = 0.699, SWB = 0.590, PCR = 0.923), demonstrating good convergent validity of the measures (Fornell and Larcker, 1981).

Discriminant validity was assessed by comparing the square root of the AVE for each latent construct with Pearson correlation coefficients calculated from composite scores obtained by summing its observed indicator scores (Fornell and Larcker, 1981). As shown in Table 4, each square root of AVE exceeded the corresponding correlation coefficients, indicating satisfactory discriminant validity. Physical activity was not included in this assessment because it was modeled as an observed variable.

**Table 4:** Fornell–Larcker matrix for the latent constructs.

Dimension	SWB	ER	PCR
SWB	0.768		
ER	0.297	0.836	
PCR	0.325	0.269	0.961

*N* = 719. PA, physical activity; ER, emotion regulation; SWB, subjective wellbeing; PCR, parent–child relationship.

### 3.2 Assessment of common method bias

Harman's single-factor test was conducted using all 67 original items included in the final analyses, including the three PARS-3 items. The unrotated principal component analysis identified four components with eigenvalues greater than 1. The first component accounted for 32.39% of the total variance, which was below the 40% threshold. This result provided preliminary evidence that common method bias was unlikely to be serious. Physical activity was excluded from the subsequent latent method factor analysis because it was represented in the structural model by a multiplicative total score and modeled as a single observed variable.

Following the latent-variable approach to method effects (Williams and Anderson, 1994), an unmeasured latent method factor test was subsequently conducted. Two nested models were estimated. In Model 1, the loadings of the additional method factor on the observed indicators of emotion regulation, subjective wellbeing, and the parent–child relationship were fixed at zero, representing a model without variance attributable to the common method factor. In Model 2, these loadings were constrained to a single freely estimated parameter. A significant reduction in the chi-square value of Model 2 relative to Model 1 would indicate that the method factor explained additional covariance among the observed indicators. Model 2 (χ^2^ = 564.169, df = 489) did not fit the data significantly better than Model 1 (χ^2^ = 564.363, df = 490), Δχ^2^ = 0.194, Δdf = 1, *p* = 0.660. These results suggest that common method bias was unlikely to pose a serious threat to the findings.

### 3.3 Correlation analysis

Pearson correlations were calculated using the observed composite scores for the four study variables. The results showed that all correlations among the four main variables were statistically significant (see Table 5). Physical activity was positively correlated with emotion regulation (r = 0.254, *p* < 0.01), subjective wellbeing (r = 0.277, *p* < 0.01), and parent–child relationship (r = 0.258, *p* < 0.01). Emotion regulation was positively correlated with both subjective wellbeing (r = 0.297, *p* < 0.01) and parent–child relationship (r = 0.269, *p* < 0.01). Subjective wellbeing and parent–child relationship were also positively correlated (r = 0.325, *p* < 0.01). In summary, all correlations were significant.

**Table 5:** Means, standard deviations, and Pearson correlations among the observed composite scores.

Variable	*M* ±*SD*	PA	SWB	ER	PCR
PA	50.154 ± 36.183	1			
SWB	75.968 ± 20.170	0.277^**^	1		
ER	36.033 ± 8.172	0.254^**^	0.297^**^	1	
PCR	11.509 ± 3.054	0.258^**^	0.325^**^	0.269^**^	1

*N* = 719. PA, physical activity; ER, emotion regulation; SWB, subjective wellbeing; PCR, parent–child relationship. ^**^p < 0.01.

### 3.4 Serial mediation analysis

#### 3.4.1 Model fit test

The AMOS 23.0 software was utilized to execute the model fit test. The results indicate that the model's absolute, incremental, and parsimony fit indices all meet or exceed the recommended criteria (Hair et al., 2019), demonstrating that the observed data have a good fit with the hypothesized model (see Table 6).

**Table 6:** Model fit indices.

Item	Indicator	Threshold value	Actual value
Absolute fit indices	CMIN/DF	< 3	1.080
	GFI	>0.90	0.951
	AGFI	>0.80	0.941
	RMSEA	< 0.05	0.011
Incremental fit indices	IFI	>0.90	0.997
	CFI	>0.90	0.997
	NFI	>0.90	0.961
Parsimony fit indices	PNFI	>0.50	0.838
	PCFI	>0.50	0.870

#### 3.4.2 Path coefficient analysis

Based on the good model fit, the path model was tested using AMOS 23.0. The results indicated that parental physical activity was positively associated with the parent–child relationship (β = 0.143, *p* < 0.001), supporting H1. Parental physical activity was also positively associated with emotion regulation (β = 0.264, *p* < 0.001) and subjective wellbeing (β = 0.213, *p* < 0.001). Furthermore, parental emotion regulation was positively associated with both subjective wellbeing (β = 0.252, *p* < 0.001) and parent–child relationship (β = 0.171, *p* < 0.001). Finally, parental subjective wellbeing was also positively associated with parent–child relationship (β = 0.237, *p* < 0.001). In summary, all six hypothesized structural paths were statistically significant. Figure 2 illustrates the tested serial mediation model. The standardized path coefficients (β) indicate the direction and magnitude of the hypothesized relationships. Positive values indicate positive associations, and larger absolute values denote stronger associations.

**Figure 2:**
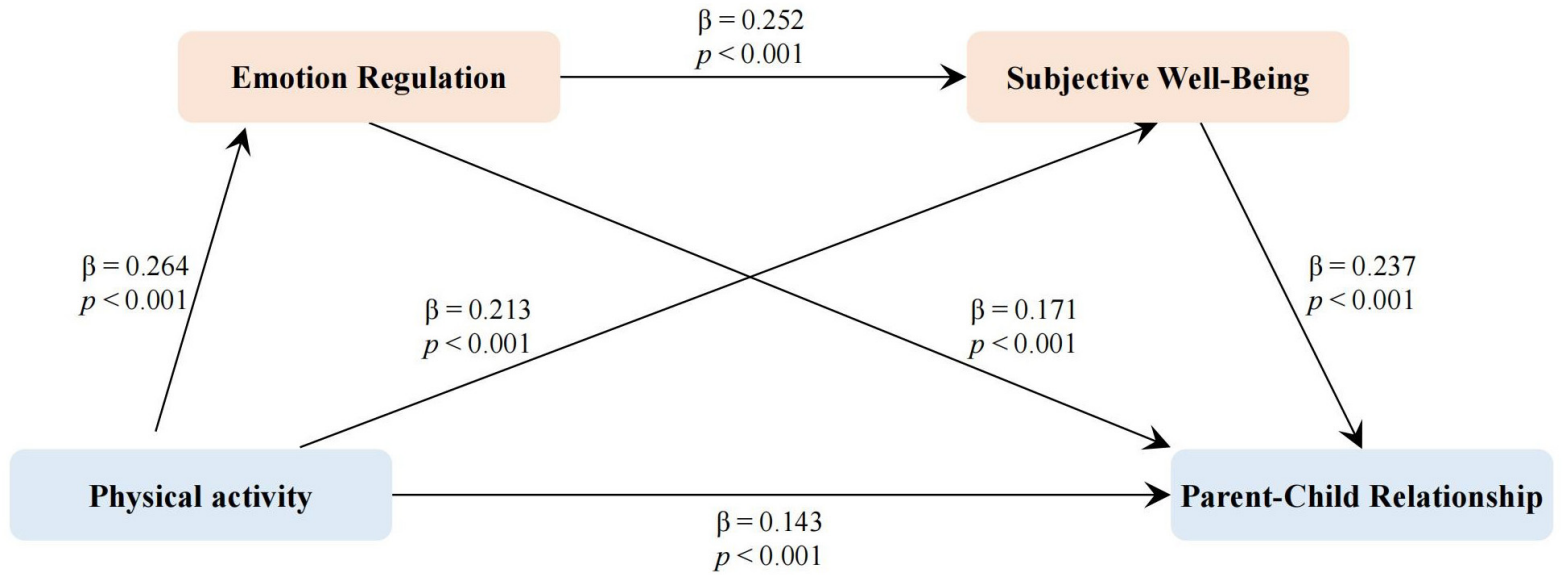
Covariate-adjusted serial mediation model. Path diagram illustrating the covariate-adjusted serial mediation model among PA, ER, SWB, and PCR. Arrows represent direct associations and are labeled with standardized path coefficients (β) and p-values. PA is associated with ER, SWB, and PCR; ER is associated with SWB and PCR; and SWB is associated with PCR. All six structural paths are statistically significant. Demographic covariates are included in the model but omitted from the figure for clarity.

Table 7 presents the standardized estimates of the paths from the demographic covariates to emotion regulation, subjective wellbeing, and the parent–child relationship. All hypothesized structural paths reported in Figure 2 remained statistically significant after adjustment for these covariates.

**Table 7:** Standardized associations of demographic covariates with the endogenous variables in the adjusted structural model.

Covariate	Comparison	ER β (*p*)	SWB β (*p*)	PCR β (*p*)
Parent gender	Male vs. female (ref.)	−0.0988 (0.0090)^*^	−0.0013 (0.9710)	0.0112 (0.7541)
Age	20–30 vs. 30–35 years (ref.)	−0.0484 (0.2184)	0.0437 (0.2500)	0.0060 (0.8712)
	>35 vs. 30–35 years (ref.)	−0.0692 (0.0848)	0.0275 (0.4785)	−0.0089 (0.8144)
Daily working hours	≤ 4 vs. 8–9 h (ref.)	0.0148 (0.7039)	0.0041 (0.9139)	−0.0201 (0.5860)
	5–7 vs. 8–9 h (ref.)	−0.0075 (0.8485)	−0.0412 (0.2769)	0.0195 (0.5999)
	>9 vs. 8–9 h (ref.)	−0.0505 (0.1943)	−0.0449 (0.2328)	−0.0320 (0.3846)
Only-child status	Only vs. non-only (ref.)	−0.0074 (0.8441)	−0.0015 (0.9674)	−0.0759 (0.0324)^*^
Child's kindergarten grade	Middle vs. junior (ref.)	−0.0085 (0.8484)	−0.0445 (0.2995)	0.0228 (0.5872)
	Senior vs. junior (ref.)	−0.0479 (0.2927)	−0.0139 (0.7513)	−0.0127 (0.7687)

*N* = 719. Values are standardized regression coefficients, with two-tailed *p* values in parentheses. ER, emotion regulation; SWB, subjective wellbeing; PCR, parent–child relationship. ^*^p < 0.05. Ref. denotes the reference category.

#### 3.4.3 Indirect association analysis

In the adjusted latent-variable SEM, the total indirect association between parental physical activity and the parent–child relationship through emotion regulation and subjective wellbeing was statistically significant (standardized indirect association = 0.111, 95% bias-corrected bootstrap CI [0.081, 0.147]).

The three specific indirect associations were examined using PROCESS Model 6 based on the observed composite scores (Hayes, 2013). Parent gender, age group, daily working hours, only-child status, and kindergarten grade were included as covariates. Indirect associations were evaluated using 5,000 bootstrap samples and 95% confidence intervals. The results revealed specific indirect associations (see Table 8). The indirect estimate through emotion regulation was 0.041 (95% CI [0.020, 0.065]) (H2). The indirect estimate through subjective wellbeing was 0.050 (95% CI [0.030, 0.074]) (H3). The specific indirect estimate for the serial pathway (PA → ER → SWB → PCR) was 0.015 (95% CI [0.008, 0.023]) (H4).

**Table 8:** Covariate-adjusted specific indirect associations of parental physical activity with the parent–child relationship.

Indirect pathway	Completely standardized estimate	95% bootstrap CI (standardized)
PA → ER → PCR	0.041	[0.020, 0.065]
PA → SWB → PCR	0.050	[0.030, 0.074]
PA → ER → SWB → PCR	0.015	[0.008, 0.023]

*N* = 719. PA, physical activity; ER, emotion regulation; SWB, subjective wellbeing; PCR, parent–child relationship. The specific indirect associations were estimated using PROCESS Model 6 based on observed composite scores and were adjusted for parent gender, age group, daily working hours, only-child status, and the child's kindergarten grade. Confidence intervals were based on 5,000 percentile bootstrap samples.

## 4 Discussion

This study identified a positive direct association between parental physical activity and the parent–child relationship. Indirect pathways were also observed separately via emotion regulation or subjective wellbeing, with an additional serial pathway involving both variables. All hypothesized pathways remained statistically significant after adjustment for the demographic covariates.

### 4.1 The direct association between PA and the PCR

This study found that parental physical activity was positively associated with the parent–child relationship (β = 0.143, *p* < 0.001). This association remained statistically significant after adjustment for parent gender, age, daily working hours, whether the child was an only child, and the child's kindergarten grade. The standardized coefficient was positive but relatively modest in magnitude, suggesting that parental physical activity represents only one of several factors potentially related to parent–child relationship quality. Recent research on parenting practices has begun to reveal the broader relevance of parents' physical activity to family life. Specifically, more physically active parents reported providing greater support for their children's participation in physical activity (Saunders et al., 2024). This pattern suggests that parents' own activity may be reflected in more supportive engagement with their children, pointing to its broader relevance for family interaction. Research on joint parent–child activity addresses the relational dimension more directly, linking participation in sports and outdoor activities with relationship closeness, although findings have varied across parent and child reports (Knoester and Randolph, 2019; Knoester and Fields, 2020). Despite their different emphases, both lines of research suggest that physical activity may be relevant to family interaction. Family systems theory offers a useful perspective for considering possible explanations for the association observed in this study.

The theory posits that an individual's behavior within the dynamic family system may define, maintain, or change their relationships with other family members (Bavelas and Segal, 1982). Based on this theory, we speculate that the observed association between parents' physical activity and the parent–child relationship may be understood in terms of two possible explanations. First, from a psychological perspective, physical activity among parents may be associated with lower levels of parenting stress and more positive emotions, which could help create a healthy family environment and foster positive and enjoyable parent–child interactions, thus potentially supporting the quality of the parent–child relationship. Second, the positive psychological states that may accompany physical activity could be associated with more patient and constructive parenting responses when parents face children's behavioral problems or parent–child conflicts. Such responses may support a positive parent–child relationship. The direct association identified in this study broadens the scope of parental physical activity research beyond individual health outcomes and activity-specific family behaviors to overall parent–child relationship quality. This finding suggests that parents' physical activity may be relevant not only to their own health but also to their relationships with their children.

### 4.2 The indirect association through cognitive emotion regulation

The significant indirect association between parental physical activity and the parent–child relationship through emotion regulation provided support for H2. This result accords with evidence that aerobic exercise is associated with better emotion regulation (Wang et al., 2024). Research on habitual physical activity also suggests that it is related to emotion regulation during stressful situations (Perchtold-Stefan et al., 2020). These findings suggest that the positive experiences associated with parents' own physical activity may help them manage physical and psychological strain. Such emotional benefits may also be reflected in their interactions with their children. The positive association between emotion regulation and the parent–child relationship observed in this study is consistent with parenting research. Parents with stronger emotion regulation tend to show greater warmth, lower hostility, and more supportive parenting behaviors. By contrast, difficulties in emotion regulation are associated with more negative parenting behaviors (Zimmer-Gembeck et al., 2022). Improvements in parents' emotion management have also been accompanied by greater satisfaction with the parent–child relationship and stronger family harmony (Fabrizio et al., 2015). Overall, the findings indicate that emotion regulation may account for part of the association between parents' own physical activity and the parent–child relationship in early childhood.

One possible explanation for the positive association between physical activity and emotion regulation involves the endocannabinoid system. Acute exercise is associated with increased circulating endocannabinoid concentrations, while the endocannabinoid system is involved in the regulation of stress and emotional responses (Viveros et al., 2007; Desai et al., 2022). Exercise-related changes in endocannabinoid activity may therefore be relevant to parents' management of negative emotions during stressful parenting situations. This interpretation concerns parents' regulation of their own emotions and therefore primarily reflects intrinsic emotion regulation rather than attempts to regulate their children's emotions (Gross, 2015). It does not identify any particular emotion regulation strategy as responsible for the indirect association. Since endocannabinoid responses were not measured in this study, this account remains a possible explanation rather than a mechanism verified by the findings.

### 4.3 The indirect association through subjective wellbeing

In this study, parental physical activity was positively associated with subjective wellbeing, and the statistically significant but modest indirect association through subjective wellbeing provided support for H3. This finding accords with previous research showing that physical activity is related to greater life satisfaction (Wiese et al., 2018) and higher levels of happiness (An et al., 2020). It is also consistent with evidence that mothers with greater life satisfaction tend to engage in more positive parenting behaviors (Song et al., 2019). Two possible explanations for this indirect association relate to parents' physical health and psychological resilience.

First, regular physical activity is associated with better cardiorespiratory and immune function, more favorable metabolic health, and better perceived physical health (Belanger et al., 2022), all of which may contribute to subjective wellbeing (Ngamaba et al., 2017). These health-related correlates provide a possible explanation for the positive association between parental physical activity and subjective wellbeing observed in this study.

Second, higher levels of physical activity have been associated with greater psychological resilience, better mental health, and stronger self-efficacy. These favorable psychological states are also related to higher life satisfaction and happiness (Guo, 2025; Peng et al., 2025). Previous studies have reported that greater psychological resilience is associated with lower parenting stress. Parents with greater resilience may also be better able to maintain a positive attitude in the face of complex parenting tasks and use more effective parent–child communication strategies (Maurović et al., 2020; Dijkstra-de Neijs et al., 2026). In summary, more favorable physical and psychological states associated with physical activity may correspond to higher subjective wellbeing among parents, which may be reflected in more positive interactions with their children.

### 4.4 The serial indirect association through ER and SWB

Beyond the separate indirect associations through emotion regulation and subjective wellbeing, the statistically significant but modest serial indirect association through both variables provided support for H4. This pattern is consistent with previous research. Emotional self-regulation may partly account for the association between physical activity and subjective wellbeing, and this link between emotion regulation and wellbeing among parents may also be relevant to parent–child relationship quality (Budiarto et al., 2020; Fuentealba-Urra et al., 2023; Shenaar-Golan et al., 2023). This study extends prior work by examining parents' own physical activity, emotion regulation, subjective wellbeing, and parent–child relationship quality within a single serial model.

Family systems theory offers one possible explanation for this pattern. Parents' behaviors and psychological states are embedded within ongoing family interactions, so the emotion regulation and subjective wellbeing associated with parents' physical activity may also be reflected in the quality of their relationships with their children (Bavelas and Segal, 1982). More adaptive cognitive emotion regulation strategies may be associated with better management of negative experiences and more favorable evaluations of life. Higher subjective wellbeing may correspond to greater patience, warmth, and constructive communication during everyday interactions with children. In this sense, cognitive emotion regulation and subjective wellbeing may jointly help account for how an individual health behavior is related to a family relationship.

A complementary explanation for the associations among physical activity, cognitive emotion regulation, and subjective wellbeing can be provided by Conservation of Resources Theory. According to this theory, the fundamental motivation of individuals is to protect and accumulate resources, including materials, conditions, energy, and personal characteristics. The threatened or actual loss of these resources may generate stress, whereas resource gains may contribute to wellbeing (Hobfoll, 1989). On the one hand, the process of physical activity may involve interaction with others, which can help individuals obtain social support and stimulate positive emotions (Huang and Wong, 2025), so that individual resources can be preserved and added to, thus creating a sense of happiness. The extant research also suggests that the regulation strategy of amplifying positive emotions can promote individuals to obtain psychological resources (Lapalme et al., 2023), which is consistent with the aforementioned view. On the other hand, in the absence of external pressures, individuals proactively engage in various strategies to accumulate resources, thereby achieving a “resource surplus” that serves as a safeguard against potential future losses (Hobfoll, 1989). As previously indicated, regular physical activity may support the accumulation of personal characteristic resources (including adaptive emotion regulation) and may contribute to attaining a “resource surplus.” For parents of young children, these resource associated with physical activity may help to cushion the loss of resources caused by parenting demands and may support the maintenance of subjective wellbeing.

## 5 Limitations

Several limitations should be noted. First, the cross-sectional design does not establish temporal order and therefore does not permit definitive causal conclusions. The use of convenience and snowball sampling in six Chinese cities may also limit the generalizability of the findings to parents in other regions or cultural contexts. Future research may expand the scope of sample selection and use longitudinal research designs to validate or refine the findings. Second, the study did not examine certain potential variables that may have influenced the results, such as parenting styles or behaviors. Although several demographic characteristics were included as covariates, residual confounding from unmeasured family and parenting factors cannot be ruled out. Subsequent studies could include a broader range of theoretically relevant covariates. Third, all focal variables were reported online by the same parent at one time point. Although Harman's single-factor test and the latent method-factor analysis did not indicate a substantial common method effect, these statistical tests cannot rule out recall bias, social desirability bias, or other method-related measurement error arising from single-informant self-reports. Future studies could use multi-informant data or more objective measures.

## 6 Conclusion

This study examined the association between parental physical activity and the parent–child relationship and investigated the indirect roles of emotion regulation and subjective wellbeing. The main conclusions are as follows:

First, parental physical activity was significantly and positively associated with the parent–child relationship.

Second, the indirect associations through emotion regulation and subjective wellbeing were both statistically significant.

Third, the serial indirect association between parental physical activity and the parent–child relationship through emotion regulation and subjective wellbeing was statistically significant.

In general, the findings suggest that emotion regulation and subjective wellbeing may partly account for the association between parental physical activity and the parent–child relationship. These associations remained statistically significant after adjustment for the measured demographic characteristics. Given their modest magnitude, parental physical activity should be regarded as one of several factors related to parent–child relationship quality.

## Data Availability

The raw data supporting the conclusions of this article will be made available by the authors, without undue reservation.
